# Clinical Long-Term Outcomes of Patient-Reported Outcomes in the Prospective Real-World Tofacitinib Response in Ulcerative Colitis Registry

**DOI:** 10.14309/ctg.0000000000000669

**Published:** 2023-12-22

**Authors:** Hans H. Herfarth, Anita Afzali, Monika Fischer, David Hudesman, Maisa Abdalla, Robert McCabe, Benjamin L. Cohen, Ryan C. Ungaro, Will Harlan, John Hanson, Gauree G. Konijeti, Steven Polyak, Timothy Ritter, Bruce Salzberg, Jennifer Seminerio, Emily English, Xian Zhang, Millie D. Long

**Affiliations:** 1 University of North Carolina at Chapel Hill, Division of Gastroenterology and Hepatology, Chapel Hill, North Carolina, USA;; 2 University of Cincinnati, Cincinnati, Ohio, USA;; 3 Indiana University, Division of Gastroenterology and Hepatology, Indianapolis, Indiana, USA;; 4 NYU Langone Medical Center, New York, New York, USA;; 5 University of Rochester, Division of Gastroenterology and Hepatology, Rochester, New York, USA;; 6 MNGI Digestive Health, Minneapolis, Minnesota, USA;; 7 Division of Gastroenterology, Icahn School of Medicine at Mount Sinai, New York, New York, USA;; 8 Digestive Health Partners, Ashville, North Carolina, USA;; 9 Atrium Health Gastroenterology and Hepatology, Charlotte, North Carolina, USA;; 10 Scripps Clinic, Division of Gastroenterology & Hepatology, La Jolla, California, USA;; 11 University of Iowa, Division of Gastroenterology and Hepatology, Iowa City, Iowa, USA;; 12 GI Alliance Research, Southlake, Texas, USA;; 13 Atlanta Gastroenterology Specialists, Atlanta, Georgia, USA;; 14 Division of Digestive Diseases and Nutrition, University of South Florida Morsani College of Medicine, Tampa, Florida, USA.

**Keywords:** tofacitinib, ulcerative colitis, inflammatory bowel diseases, patient-reported outcomes, safety

## Abstract

**INTRODUCTION:**

We previously reported the results of tofacitinib induction therapy in the prospective multisite US real-world Tofacitinib Response in Ulcerative Colitis registry. We now assessed patient-reported outcomes (PROs) and predictors of success during tofacitinib maintenance therapy.

**METHODS:**

Tofacitinib Response in Ulcerative Colitis included 103 patients with refractory ulcerative colitis (UC); 67% had failed ≥ 2 biologics. Patients reported the Simple Clinical Colitis Activity Index (SCCAI), Patient-Reported Outcome Measurement Information System measures for anxiety, depression, social satisfaction, and adverse events between weeks 8 and 52 using a web-based system. Paired *t* test and *P* for trend were used to compare changes in PRO measures over time. Bivariate analyses and logistic regression models were used to determine factors associated with response (SCCAI <5) or remission (SCCAI <2) at week 52.

**RESULTS:**

Of 103 patients, 82.5% entered the maintenance phase and 43.7% remained on tofacitinib at week 52. Tofacitinib de-escalation to 5 mg BID occurred in 15% of patients. At week 52, 42.7% and 31.1% of all patients reported an SCCAI <5 and SCCAI ≤2, respectively. Normalization of bowel frequency, rectal bleeding, and urgency occurred in 79%, 61%, and 48% of patients remaining on maintenance therapy. Social satisfaction improved significantly (*P* < 0.001), while anxiety and depression scores only numerically improved. No consistent predictors for tofacitinib long-term treatment efficacy were identified, and safety findings were consistent with the known safety profile of tofacitinib.

**DISCUSSION:**

Tofacitinib is an effective maintenance therapy in patients with refractory UC. Dose reductions infrequently occurred during maintenance. Unmet needs in UC maintenance include improvement of urgency and psychosocial factors (NCT03772145).

## Graphical abstract

**Figure:**
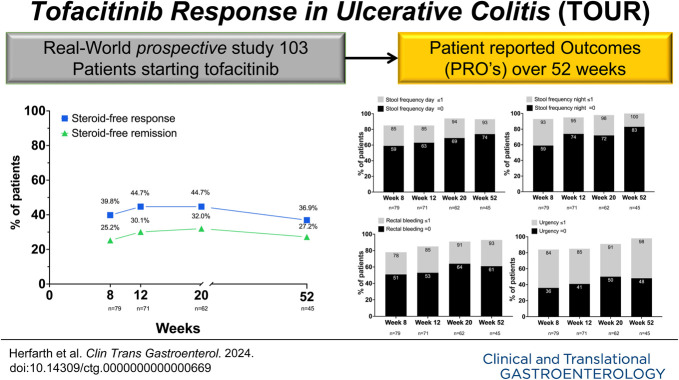

## INTRODUCTION

Ulcerative colitis (UC) is a chronic recurrent inflammatory bowel disease (IBD) currently treated with a variety of treatment options, including aminosalicylates, corticosteroids, thiopurines, calcineurin inhibitors, anticytokines, anti-integrins, and small-molecule Janus kinase (JAK) inhibitors (1,2). Tofacitinib is an oral small-molecule JAK inhibitor approved for moderate-to-severe UC based on short-term and long-term clinical efficacy and safety in the oral clinical trials for tofacitinib in ulcerative colitis clinical trial program (3,4). However, it is recognized that randomized controlled trials, which constitute the gold standard for regulatory approval, inadequately reflect the patient population in everyday clinical practice. In addition, because regulatory trials commonly perform rerandomization of only responders after the induction phase, the actual efficacy of nearly all recently approved IBD drugs is inflated (5). So-called real-world observational studies extend beyond the scope of the defined regulatory questions and provide data about short-term and long-term follow-up for the safety and effectiveness of drugs in routine clinical practice. The regulatory authorities such as the US Food and Drug Administration (US FDA) and the European Medicines Agency (EMA) increasingly incorporate real-world evidence studies in their program (6,7). So far, most of the tofacitinib real-world data have been retrospective assessments, which may introduce biases (8). In addition, most prior studies focus on induction data, with limited data available on the long-term outcomes of tofacitinib therapy beyond 6 months (9,10).

The US FDA and EMA recommend reducing the dose of tofacitinib from 10 to 5 mg bid after 8–16 weeks of therapy. This recommendation for risk minimization is based on the potentially increased risk of major adverse cardiovascular events, venous thromboembolism, cancer, and severe infections on higher doses of tofacitinib (11). So far, real-world data about the frequency of dose reduction are sparse, particularly because tofacitinib in the United States is only approved after anti–tumor necrosis factor (TNF) failure, which may constitute a patient population with more severe disease.

The Tofacitinib Response in UC (TOUR) registry is a prospective multicenter cohort of adult patients who initiated tofacitinib therapy for moderate-to-severe UC. The predefined outcomes of the study included assessments of the short-term and long-term efficacy and safety of tofacitinib. In contrast to the previously published cohorts, the TOUR focuses on prospectively collected patient-reported outcomes (PROs) and the National Institute of Health Patient-Reported Outcome Measurement Information System (PROMIS) measures to evaluate symptom improvement. We have recently reported the induction data, finding a very early onset of efficacy at day 3 and a steroid-free remission rate of 29% around week 8 (12). In this study, we report the prospectively evaluated maintenance efficacy and safety data, including PROs for depression, anxiety, and social satisfaction, between weeks 8 and 52 of tofacitinib therapy.

## METHODS

### Study setting and design

The TOUR study is an observational prospective cohort study conducted in 14 sites across the United States (NCT03772145). Patients were enrolled if there was an intent to start tofacitinib for moderate-to-severe UC. Enrollment in the cohort occurred between February 2019 and continued through July 2022, and we previously reported results of tofacitinib induction therapy through week 8 (12). We now describe the outcomes of the maintenance phase of the complete cohort between weeks 8 and 52.

### Inclusion criteria and tofacitinib dosing

Adult patients (older than 18 years) with UC established by usual endoscopic, histologic, and radiologic criteria who started tofacitinib as part of the standard of care and agreed to be followed up by the site for at least 12 months were eligible for inclusion. To adhere to the real-world setting, there were no prespecified inclusion or exclusion criteria for participation in the study; specifically, we did not use prespecified disease activity criteria. The only exclusion criterion was the inability to use the English language and/or lack of internet access. Because this was a real-world observational study, there were no predefined drug or dosing regimens. The doses of tofacitinib and steroid tapering were at the discretion of the local investigators. Compliance with individual drug intake was not evaluated in the context of this study.

### Data collection and questionnaires

As previously described, the multicenter TOUR study used a novel electronic web-based PRO system, with direct data capture from patients, while still obtaining site-based objective clinical data (12). The PRO system facilitated adherence to the protocol, and we achieved high response rates of >90% at each data point. At prespecified time points during the tofacitinib maintenance therapy at weeks 8, 12, and then every 8 weeks until week 52, specific questionnaires including the Simple Clinical Colitis Activity Index (SCCAI) and PROMIS symptom scales for depression, anxiety, and social satisfaction were sent electronically to the participating patients. The SCCAI includes 6 variables: bowel frequency during the day and night, the urgency of defecation, blood in the stool, general well-being, and extracolonic manifestations of UC; the combined score can range from 0 to 19 (13). We defined an SCCAI score of <5 as a response, which includes mild disease activity or no disease activity during evaluation, and a score ≤2 as remission during evaluation (14,15). PROMIS symptom scales were collected at the start of therapy, weeks 8 and 12, and then every 8 weeks until week 52. These scales are standardized to the general population through a T score of 50 and an SD of 10 (16). Higher T scores are associated with more of the domain; thus, higher T scores for depression and anxiety are worse, whereas higher T scores for social satisfaction are better.

### Outcomes and definitions

The primary outcome for the maintenance phase was the proportion of patients with a response (defined as an SCCAI <5, which reflects mild activity or no disease activity during evaluation as outlined earlier) at week 52 (17,18). The secondary outcome was clinical remission at week 52, defined as an SCCAI score of ≤2 (15,19). In addition, the following events were recorded: new onset of shingles, rate of shingles vaccination, infections resulting in the need for antibiotic therapy, hospitalizations, and UC-related surgeries. If tofacitinib was discontinued, the reason for discontinuation was captured, and patients were censored as a failure.

### Statistical analysis

Continuous variables were summarized using mean values and SD. Comparisons used the Student *t* test or Wilcoxon rank sum test. Repeated continuous measures were compared using paired *t* test and *P* for trend. Categorical variables were expressed as proportions and compared using the χ^2^ test or Fisher exact test where appropriate. The denominator for response and remission to tofacitinib therapy at the conclusion of 52 weeks consisted of all patients starting tofacitinib therapy at baseline. For evaluation of the SCCAI subscores and PROMIS measures, the denominator consisted of the number of patients still in the study and submitting the questionnaires at the predefined time points.

Multivariate analyses were performed using logistic regression to determine factors predictive of response and remission. Variables considered *a priori* to be related to response or remission were entered into the model. SAS (SAS Institute, Cary, NC) was used for all analyses.

### Ethical considerations

The protocol was approved by each participating center's institutional review board or independent ethics committee. All patients provided written informed consent.

## RESULTS

**Figure 1.**
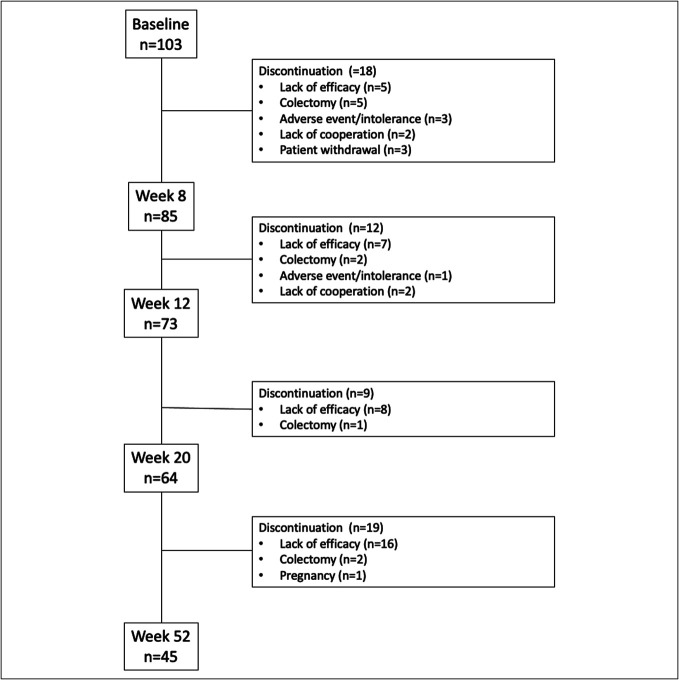
Study flow diagram with timing and reasons for tofacitinib withdrawal.

One hundred three patients were enrolled in the TOUR registry (Table 1). Of them, 82.5% (85/103) of patients were still actively treated at week 8, and 43.7% (45/103) completed the maintenance period at week 52 (Figure 1). Their baseline demographics and disease characteristics are summarized in Table 1. Failure of ≥2 biologics had occurred in 67.0% (69/103) of patients, and 64.1% (66/103) were on concomitant steroids at baseline. Of the enrolled patients, 84.5% (87/103) reported clinically active disease with an SCCAI >2 at the start of tofacitinib. Endoscopic disease activity was available in 97.1% (100/103) of patients, and moderate-to-severe endoscopic severity (Mayo score 2 or 3) was recorded in 89.0% (89/100). Drug persistence, defined as continuous tofacitinib therapy and remaining in the TOUR registry over 52 weeks, was 43.7% (45/103).

**Table 1.** Characteristics of patients in the TOUR study (n = 103)

Age (yr, mean, range)	38.0	18–81
Sex (m: n; %)	58	56.3%
Duration of disease (yr; mean, range)	8.6	0–50
BMI (kg/m^2^); (mean, range)	25.5	14.8–43.9
Race (n, %)		
White	87	84.5%
Black/African American	6	5.8%
Asian	2	1.9%
Other	5	4.9%
Unknown	3	2.9%
Current smoker	2	1.9%
Site of disease (Montreal classification)		
E1	7	7%
E2	45	44%
E3	51	50%
Prior medication use (n, %)		
Mesalamine	97	92.2%
Steroids (at baseline; wk 0)	66	64.1%
Azathioprine/6-MP	46	44.7%
Methotrexate (oral or sc)	22	21.4
Vedolizumab	63	61.2%
Ustekinumab	8	7.8%
Anti-TNF	91	94.8%
Total no. of prior biologics		
0	3	2.9%
1	31	30.1%
2	38	36.9%
3	24	23.3%
4	7	6.8%
SCCAI >2	87	84.5%
SCCAI ≤2	16	15.5%
Mayo endoscopy score^a^		
0	4	3.9%
1	7	6.8%
2	37	35.9%
3	52	50.5%
Unknown	3	2.9%

BMI, body mass index; SCCAI, Simple Clinical Colitis Activity Index; TNF, tumor necrosis factor; TOUR, tofacitinib response in ulcerative colitis; UC, ulcerative colitis.

a Endoscopy score before tofacitinib initiation (most recent colonoscopy or sigmoidoscopy reported in the system).

### Mild disease activity and remission in the maintenance period

**Figure 2.**
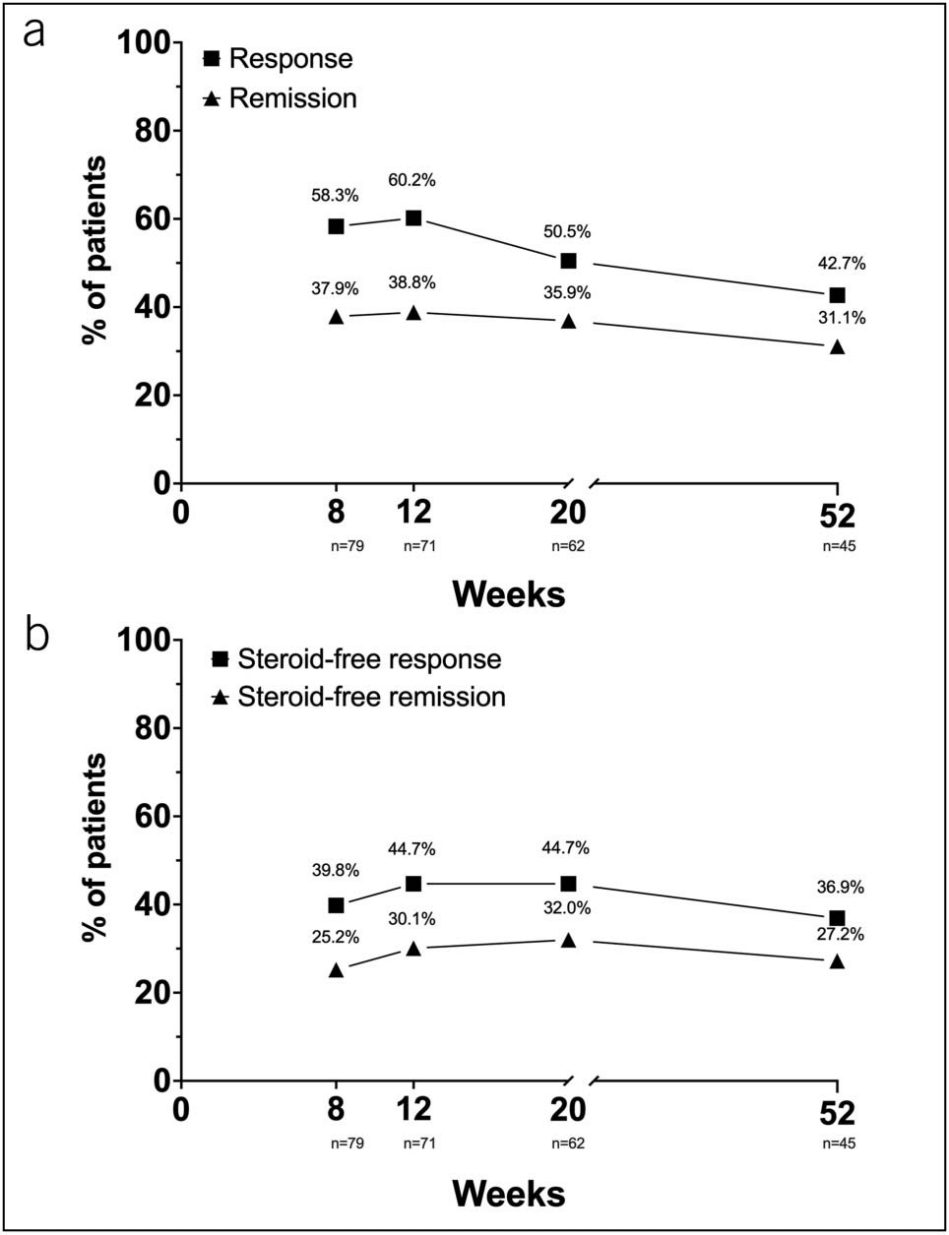
(**a**) Proportions of patients with response, defined as mild or no disease activity (SCCAI <5) and remission (SCCAI ≤2) between week 8 and week 52 (n = 103). (**b**) Proportions of patients with steroid-free response, defined as mild or no disease activity (SCCAI <5) and remission (SCCAI ≤2) between week 8 and week 52 (n = 103). At weeks 8, 12, 20, and 52, 79/85 (92.9%), 71/73 (97.3%), 62/64(96.9%), and 45/45 (100%) patients, respectively, reported the SCCAI scores in the visit window. SCCAI, Simple Clinical Colitis Activity Index.

At week 52, 42.7% (44/103) and 31.1% (32/103) of patients who initially started tofacitinib were in response (SCCAI <5) or in remission (SCCAI <2) (Figure 2a). Steroid-free response and remission occurred in 36.9% (38/103) and 27.2% (28/103), respectively (Figure 2b). The highest proportion of patients in response or remission occurred at week 12; the proportions for both outcomes gradually declined over the maintenance period. Of 43 patients with moderate-to-severe disease activity (SCCAI ≥5) at week 8, who continued on tofacitinib therapy throughout 52 weeks, 25.6% (11/43) reported a response (23.2%; 10/43) or remission (2.3%; 1/43) at week 52. Of the 11 patients with a delayed response or remission, 3 (all responders) were not steroid-free at week 52.

### SCCAI subscores

**Figure 3.**
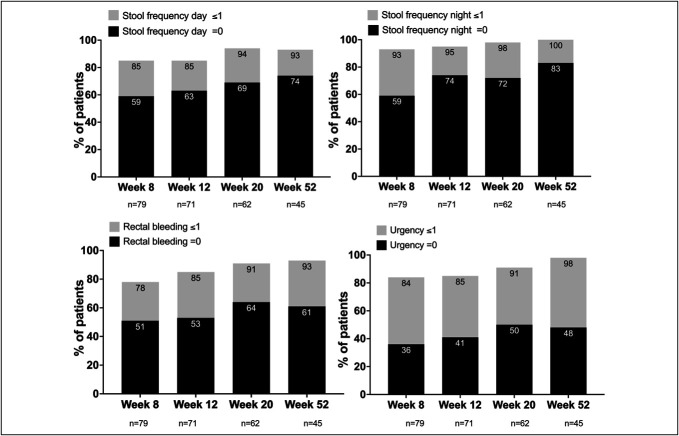
Percentage of patients with SCCAI subscores of 0 and ≤1 for daily stool frequency, nightly stool frequency, rectal bleeding, and urgency at weeks 8, 12, 20, and 52. At weeks 8, 12, 20, and 52, 79/85 (92.9%), 71/73 (97.3%), 62/64 (96.9%), and 45/45 (100%) patients, respectively, reported the SCCAI scores in the visit window. SCCAI, Simple Clinical Colitis Activity Index.

SCCAI subscores of stool frequency during day and night, rectal bleeding, and urgency improved over the maintenance period, and more than 90% of patients reported no or mild symptoms for each category at week 52 (Figure 3). Combining day and night bowel frequency, 79% of patients reported a subscore of 0 at week 52 compared with 59% at week 8. The more granular analysis of day and night bowel frequency revealed that 74% and 83% of patients reported a subscore of 0 for bowel frequency during the day and night compared with 59% at week 8. The subgroup of patients reporting no blood in the stool between weeks 8 and 52 increased from 51% to 61%. Whereas urgency scores of 0 increased from 36% in week 8 to 48% in week 52, at no time points, >50% of patients reported complete resolution of urgency.

### Predictors of response or remission at week 52

Lack of steroids at week 8 or response or remission at week 12 was significantly associated with response or remission at week 52 (Table 2). By contrast, there was no relationship between response or remission on tofacitinib therapy at week 52 and the number of previously failed biologics, the severity of endoscopic inflammation at baseline (as measured by the endoscopic Mayo score), response or remission at week 8, or lack of steroids at week 12.

**Table 2.** Clinical factors associated with mild disease activity or remission at week 52

	Corticosteroid use at wk 8 n = 85^a^			Response at wk 12 (SCCAI <5) n = 73^b^			Remission at wk 12 (SCCAI ≤2) N = 73^b^		
	Yes n = 25	No n = 57	*P* value	Yes n = 57	No n = 14	*P* value	Yes n = 38	No n = 33	*P* value
Response at wk 52 (SCCAI < 5) n = 44	36%	60%	0.05	70%	29%	0.004	71%	50%	0.01
Remission at wk 52 (SCCAI ≤2) n = 32	24%	46%	0.07	51%	21%	0.05	53%	35%	0.03

Response was defined as mild or no clinical disease activity at the time point of evaluation and remission as no disease activity at the time point of evaluation.

Other factors not associated with mild disease activity or remission at week 52 included the following: Mayo endoscopic score 3 vs ≥3 at baseline, <2 vs ≥2 previous biologic failures before initiation of tofacitinib, mild disease activity (SCCAI <5) or remission status (SCCAI ≤2) at week 8 or corticosteroid use at week 12.

SCCAI, simple clinical colitis activity index.

a 85 patients in the study, but for 3 patients, steroid use was not known at week 8.

b 73 patients in the study, but for 2 patients, SCCAI values were not known in the predefined time frame week 12.

### Tofacitinib dose reductions

Tofacitinib dose reduction was initiated at the treating physician's discretion. All patients received 10 mg of tofacitinib bid during the initial 8 weeks of treatment; no dose reduction occurred during this time. Between weeks 8 and 20 and during the whole study duration, 11.8% (10/85) and 15.3% (13/85) of patients, respectively, de-escalated tofacitinib dose during maintenance therapy. After reduction, 46.1% (6/13) of the de-escalated patients remained on 5 mg bid (n = 5) or 11 mg (n = 1) daily, 23.1% (3/13) discontinued because of lack of efficacy, and 30.8% (4/13) of patients escalated the dose of tofacitinib back to 10 mg twice daily (n = 3) or 22 mg (n = 1) before week 52.

### PROMIS measures

At week 8, 46%, 32%, and 38% met the criteria for anxiety, depression, and poor social satisfaction scores, respectively. By week 52, 40% met the criteria for anxiety and 29% for depression. While numerically improved, these changes did not meet statistical significance. By contrast, of the initial 38% of patients reporting poor social satisfaction, only 18% reported poor social satisfaction at week 52 (*P* < 0.0001 for trend).

### Adverse events and discontinuations

Nineteen patients (22.3; 19/85) reported 29 episodes of antibiotic treatments for infectious complications between weeks 8 and 52 (Table 3). Indications for antibiotic therapy were most often gastrointestinal-related (suspicion of infection or colitis symptoms), followed by upper respiratory tract infections. Four patients were hospitalized because of UC flare; one patient was hospitalized 3 times between weeks 8 and 52 and another twice between weeks 38 and 46 (Table 3). One patient was hospitalized because of ectopic pregnancy. During the maintenance treatment period, no new onset of shingles was reported. At baseline, 26.2% (27/103) had received shingles vaccination (Shingrix). The percentage of completely vaccinated patients in the registry increased over the trial period (week 8: 43.5%; 37/85); week 52 (53.3%; 24/45). No thromboembolic events were reported during the maintenance period.

**Table 3.** Adverse events between weeks 8 and 52

Adverse event	Patients		Events
	n	%	
Hospitalization due to UC flare	4	4.7	7
Hospitalization other^a^	1	1.2	1
Antibiotics	19	22.3	29
Colitis/GI infection			11
Upper respiratory tract infection			7
Urinary tract infection			3
Skin infection			3
Other			5

GI, gastrointestinal; UC, ulcerative colitis.

a Hospitalization due to ectopic pregnancy.

Reasons for discontinuation of tofacitinib are listed in Table 4. Of the 85 patients entering the maintenance period at week 8, 47% (40/85) discontinued tofacitinib. Most discontinuation occurred because of a lack of efficacy (36.5%, 31/85). Five patients underwent colectomy during maintenance (5.9%; 5/85). Tofacitinib therapy was stopped in 1 patient (1.2%; 1/85) because of adverse events reported as chills, fever, and painful mouth sores. One patient became pregnant during the maintenance period. She stopped tofacitinib but had a miscarriage due to an ectopic pregnancy with the need for hospitalization. During maintenance, 2.4% (2/85) did not cooperate with survey completion and were withdrawn from the registry.

**Table 4.** Reason for discontinuation of therapy during maintenance therapy with tofacitinib between weeks 8 and 52

Study withdrawal reason	n	%
Discontinuation of tofacitinib due to lack of efficacy	31	77.5%
Surgery (colectomy)	5	12.5%
Lack of patient's cooperation with survey completion	2	5.0%
Discontinuation of tofacitinib due to intolerance or adverse event	1	2.5%
Other^a^	1	2.5%

a Patient became pregnant and stopped the medication.

## DISCUSSION

The TOUR multicenter registry is a unique prospective real-world cohort study, which describes a therapy refractory patient population with UC, with two-thirds having failed at least 2 biologics and more than 60% on concomitant steroids at baseline. Including 5 patients from the previously reported 8-week induction period, the 1-year colectomy rate in the TOUR was 9.7%, which underscores the disease severity of the included patients. At week 52, nearly one-third of all patients who initially started tofacitinib were in steroid-free remission. Patient-reported daily bowel frequency and bleeding continued to improve, and between 60% and 80% reported normalization of these indexes at week 52. Urgency also improved, but not as consistently. Slightly less than 50% of patients were urgency-free after 52 weeks. Only a minority of patients in the TOUR underwent tofacitinib dose reduction in the maintenance period, and more than half of these patients had a recurrence of symptoms. During maintenance therapy, the PROMIS domains of anxiety and depression numerically improved, whereas patients reported a significant enhancement in social satisfaction.

The observed remission rates in the TOUR of 38%, 36%, and 31% at weeks 8, 20, and 52, respectively, are overall comparable with currently available data from a recent meta-analysis of 23 retrospective and 3 prospective real-world studies, reporting remission rates of approximately 30% (95% confidence interval [CI] 22%–37%) at week 8, 32% (95% CI 28%–37%) at 6 months, and 38% (95% CI 34%–42%) around year 1 of therapy (10). The later published real-world effectiveness and safety of tofacitinib for moderate-to-severely active ulcerative colitis (REMIT-UC) study, a retrospective study with the largest patient population of 375 patients in Canada, was not included in the abovementioned meta-analysis. REMIT-UC reported remission rates of approximately 35% between weeks 12 and 52 and response rates similar to the reported proportions of patients in response with mild or no disease activity in the TOUR of 64% at week 12 (TOUR: 60%), 50% at week 24 (TOUR: 50% week 20), and 45% at week 52 (TOUR: 43%) (20). Due to the prospective approach of TOUR, we could additionally reliably assess steroid-free remission rates, which were slightly lower (−8.7% week 12, −3.9% week 20, and −3.9% week 52) but in the same range as the overall remission rates, indicating the robust efficacy of tofacitinib in patients responding to this drug. As discussed in the previously published 8-week results of the TOUR, inclusion in the TOUR registry was not dependent on predefined disease activity, and approximately 16% of patients were in remission at the start of tofacitinib therapy (12). Most of these patients were on concomitant steroid therapy, which was most likely initiated in the setting of a lengthy drug approval process.

Similar to previous studies, no compelling predictors for long-term treatment success could be detected in the TOUR (20,21). We found a statistically significant association between lack of steroids at week 8 and response or remission at week 52, but no association was found with lack of steroids at week 12 and response or remission at week 8. The number of previously failed medications and the severity of endoscopic inflammation were not associated with later treatment success, stressing the urgent need to find reliable biomarkers for predicting the therapeutic response of specific therapies in the individual patient.

Due to the repeated evaluation of disease activity PROs in the SCCAI, we could show that stool frequency during day and night and rectal bleeding continued to improve during the maintenance therapy. More than 90% of patients had only mild symptoms in these categories, whereas 60%–80% had no symptoms at week 52. In addition, patient-reported urgency improved, and nearly 100% of patients reported only mild symptoms, but complete resolution of urgency could only be achieved in less than 50% between weeks 12 and 52. We did not record the use of antidiarrheals, which could have affected the urgency reporting. Only recently, data from the mirikizumab registry program showed an improvement or resolution of urgency in 49% and 65%, and 22% and 43% at weeks 12 and 52, respectively (22). A post hoc analysis of the upadacitinib phase 3 program demonstrated that 58% and 55% of patients on 15 mg of upadacitinib and 60% and 46% of patients on 30 mg of upadacitinib reported no urgency at week 12 and 52, respectively (23). The SCCAI scale used in the TOUR, the Urgency Numeric Rating Scale used in the mirikizumab program, and the urgency evaluation applied in the upadacitinib program defining grades of urgency over a predetermined 3-day period cannot be directly compared and further signify the need for a standardized evaluation of urgency in clinical studies. However, all 3 studies indicate that a substantial portion of patients do not experience resolution of urgency, which, from the patient perspective, aside from disease control and prevention of cancer, is the third most crucial aspect of UC medical management (24). The persistence of urgency may also be due to the recently described impact of histologic healing on rectal compliance, which is only comparable with healthy controls once complete histologic resolution of inflammation is achieved (25).

Only 15% of TOUR patients de-escalated tofacitinib in the maintenance period, and most of them failed the lower dose and had re-escalated to the higher dose again or switched out of class. Dose reductions in other real-world studies occurred in more than 60%–70% of patients (Canada, UK, Spain) (20,26,27). The low de-escalation rate in the TOUR seems counterintuitive to the recent US FDA and EMA recommendations. One explanation may be the high risk of colectomy in this refractory patient population and the concerns of therapeutic failure due to low drug levels. Another explanation is that providers in the TOUR were aware of the results of the phase 3b/4, double-blind, randomized RIVETING study (11,28). This trial demonstrated that patients with more severe endoscopic inflammation and previous failure of anti-TNF therapy harbor a significantly higher risk of relapse on lower doses of tofacitinib (28). The severity of endoscopic inflammation and previous exposure to anti-TNF have also been identified as risk factors of losing response to lower doses of tofacitinib by the real-world Canadian REMIT-UC study (20).

As previously reported for the TOUR induction data, approximately half of the included population met clinical criteria for anxiety, depression, and reduced social satisfaction at baseline as evaluated by the PROMIS scales (12). While anxiety and depression scores numerically improved over the maintenance period, approximately 40% and 30% of patients reported mild-to-moderate anxiety and depressive symptoms at week 52. Only social satisfaction significantly improved; less than one-fifth of the patients reported reduced social satisfaction at week 56 compared with nearly 40% at week 8. Active IBD has been associated with reduced satisfaction with life, and our cohort showed improved social satisfaction over the maintenance period as disease activity improved (16,29). The lack of significant improvement of anxiety and depression is most likely due to many concomitant factors in the therapy refractory TOUR population. Due to the pragmatic approach, we did not collect data about the use of antidepressants, anxiolytics, or socioeconomic factors (e.g., unemployment); however, most of the TOUR follow-ups occurred during the COVID-19 pandemic, which most likely affected these PROs. A recent meta-analysis including 77 studies in patients with IBD found a pooled prevalence of anxiety symptoms of 32.1% (95% CI 28.3–36.0) and a pooled prevalence of depression symptoms of 25.2% (95% CI 22.0–28.5) (30). The reported prevalence of depression in IBD varies based on the type of measurement. E.g., using the Public Health Questionnaire-8 assessment, Kochar et al (31) reported rates of depression of 32% in UC and 38% in CD. Depression and anxiety are also associated with a more severe disease course, including higher frequency of steroid treatments, hospitalizations, and switches of therapeutic regimen (31,32). Our findings of improved but persistent symptoms of anxiety and depression support the recent recommendation for IBD providers to incorporate overall emotional wellness as a treatment goal of IBD management (33). In fact, the most recent American College of Gastroenterology guideline provided a key concept statement that “patients with UC should be screened for coexistent anxiety and depressive disorders, and when identified, patients should be provided with resources to address these conditions.” (2).

No severe infections requiring hospitalization occurred during the maintenance period. Nearly all hospitalizations occurred because of disease exacerbation except one because of a complication of an ectopic pregnancy. Herpes zoster, a known adverse event of tofacitinib therapy, occurred overall in both induction and maintenance in 3% of the TOUR population, comparable with the rate in other studies (9,34). All cases of shingles in TOUR were previously reported, and no further new cases of Herpes zoster were observed in the maintenance period (12). In TOUR, approximately one-fifth of patients were fully vaccinated at the start of tofacitinib therapy; the vaccination rates increased over time to slightly above 50%. This vaccination rate is still significantly below the 80% vaccination rate reported in the Canadian Real-World REMIT-UC study (20). However, in the Canadian study, vaccination data were unavailable for roughly 30% of the included patients, and thus, the real vaccination rate may have been overreported. On October 20, 2021, the advisory committee on immunization practices recommended 2 doses of the recombinant zoster vaccine to prevent herpes zoster in adults aged 19 years or older who are or will be immunosuppressed because of disease or therapy (35). This recommendation likely accelerated vaccination later in our study period.

A recent post hoc analysis of the subpopulations in the ORAL Surveillance, an event-driven clinical trial of risk-enriched patients with rheumatoid arthritis, identified subpopulations with a different relative risk of predefined adverse events with tofacitinib vs tumor necrosis factor inhibitors (TNFis) (36). Patients aged 65 years and older or ever smokers had an increased risk of malignancies (excluding nonmelanoma skin cancer), major adverse cardiovascular events, myocardial infarction, venous thromboembolism, and all-cause death with tofacitinib vs TNFis and were defined as “high risk.” By contrast, patients younger than 65 years and never smokers were considered “low risk” because there was no detectable risk increase with tofacitinib vs TNFis. Thromboembolic events or deep venous thrombosis were not reported during the TOUR induction or maintenance period. However, the TOUR study population included less than 7% of patients in the abovementioned high-risk age range and less than 3% of current smokers (we did not evaluate for “ever smoking”). Five thromboembolic events and 1 deep venous thrombosis, all occurring in patients on 10 mg of tofacitinib bid, have been reported in the UC study program, which included >1,100 patients (37). Worldwide postmarketing safety surveillance experience with tofacitinib in UC also suggests a relative risk of 1.26 for vascular disorders, a term that includes thromboembolic events; however, the pathophysiological mechanism underlying this adverse event is not entirely elucidated (38).

This study has several limitations. Selection bias may have occurred because patients were required to have access to the internet for the data entries. Second, due to the pragmatic trial design, we did not collect laboratory data, including C-reactive protein or calprotectin, and did not require regular endoscopic evaluations. In fact, only a small number of patients underwent endoscopic reevaluation during the maintenance period, which may have been partly due to the ongoing COVID pandemic during most of the study duration. However, the SCCAI has been shown to correlate well with endoscopic disease activity (39), and clinically relevant endpoints such as remission (40). Thus, we assume that most of the patients in clinical remission were as well in endoscopic remission at week 52.

In conclusion, the TOUR maintenance study provides the first prospective real-world evaluation of the long-term impact of tofacitinib on PROs in the United States. The study shows the impact of a JAK inhibitor therapy not only on bowel frequency and bleeding but also on the long-term outcomes of urgency and PROMIS measures of anxiety, depression, and social satisfaction. Whereas long-term therapy with tofacitinib positively affects social satisfaction, the results also indicate that anxiety and depression are persistent problems in the patient population with moderate-to-severe UC. Future research needs to define, incorporate, and address urgency as an essential patient symptom aside from the classic symptoms of bowel frequency and bleeding.

## CONFLICTS OF INTEREST

**Guarantor of the article:** Hans H. Herfarth, MD, PhD, FACG.

**Specific author contributions:** H.H.H. and M.D.L.: conceptualization and execution of the study, analysis, and interpretation of data and cowrote the manuscript. E.E.: coordination of study sites, supervision of data collection, and revised and critically reviewed the manuscript for intellectual content and accuracy. X.Z.: data analyses and interpretation of data and revised and critically reviewed the manuscript for intellectual content and accuracy. A.A., M.F., M.A., R.M., B.L.C., R.C.U., W.H., J.H.; G.G.K., S.P., T.R., B.S., and J.S.: patient recruitment and data acquisition and revised and critically reviewed the manuscript for intellectual content and accuracy. All authors approved the final version of the article.

**Financial support:** This registry was funded by Pfizer Inc.

**Potential competing interests:** H.H.H. has served as a consultant to Alivio, AMAG, BMS, Boehringer, ExeGI, Finch, Fresenius Kabi, Galapagos, Gilead, Janssen, Lycera, Merck, Otsuka, Pfizer, PureTech, Seres, and Ventyx and received research support from Artizan Biosciences, Allakos, NovoNordisk, and Pfizer. A.A. has served as a consultant for AbbVie, Takeda, Janssen, Bristol Myers Squibb, Pfizer, Eli Lilly, Gilead, DiaSorin, and TLL Pharmaceuticals and as a speaker for AbbVie, Takeda, Janssen, Bristol Myers Squibb, and Pfizer. M.F. has served as an advisory board member or consultant for AbbVie, Bristol Myer Squibb, Pfizer, Eli Lily, Takeda, Janssen, and Scioto and as a DSMB member for Rebiotix. D.H. has served as a consultant for Abbvie, BMS, Fresenius Kabi, Janssen, Pfizer, Prometheus, Takeda, and UCB and has received research support from Janssen and Pfizer. M.A. has no conflicts. R.M. has served as an advisory board member of BMS. B.L.C. has served as an advisory board member or consultant for Abbvie, Celgene-Bristol Myers Squibb, Lilly, Pfizer, Sublimity Therapeutics, Takeda, TARGET RWE; CME Companies: Cornerstones, Vindico; speaking: Abbvie; and educational grant: Pfizer. R.C.U. has served as a consultant/advisory board member for AbbVie, Bristol Myers Squibb, Janssen, Pfizer, and Takeda; received research support from AbbVie, Boehringer Ingelheim, Bristol Myers Squibb, Lilly, and Pfizer; and is funded by a National Institutes of Health (NIH) K23 Career Development Award K23KD111995-01A1. W.H. has no conflicts. J.H. has served an advisory board member for Bristol Myers Squibb and for the Speakers Bureau of AbbVie and Pfizer. G.K. has served as a consultant to Pfizer and ProciseDx and for the speakers' bureau of Takeda and Lilly. S.P. has served as an advisory board member or consultant to Pfizer and Takeda and received research support from Gilead, AbbVie, Seres, Bristol Myer Squibb, Pfizer, and Takeda. T.R. has served on advisory boards for Abbive, Arena, Boeeringer Ingleheim, Bristol Myers, Ferring, Genentech, Gilead, Intercept, Iterative Scopes, Janssen, Lilly, Pfizer, Prometheus, and Takeda and as a speaker for Abbvie, Bristol Myers, Janssen, Pfizer and Takeda. B.S. has no conflicts. J.S. has served as a consultant for AbbVie, UCB, Takeda, Janssen, Pfizer, BMS, and Prometheus and has received research support from Takeda. E.E. has no conflicts. X.Z. has no conflicts. M.D.L. has served as a consultant for AbbVie, Takeda, Janssen, Pfizer, Lilly, Genentech, Roche, BMS, Target RWE, and Prometheus and has received research support from Pfizer, Takeda, and Lilly.

**Study Highlights**

**WHAT IS KNOWN**

✓ Short-term efficacy and safety of tofacitinib in the real-world study setting

**WHAT IS NEW HERE**

✓ Long-term efficacy and patient-reported outcomes over 1 year in a real-world study in the setting of prospectively collected data.
✓ Persistence of tofacitinib therapy over 1 year.
✓ Frequency of tofacitinib dose reduction during maintenance therapy.
✓ Long-term patient-reported outcomes of bowel frequency, bleeding, urgency, Patient-Reported Outcome Measurement Information System measures, and tofacitinib adverse events.

## Supplementary Material
