# Supplementary material for: Clinical Long-Term Outcomes of Patient-Reported Outcomes in the Prospective Real-World Tofacitinib Response in Ulcerative Colitis Registry
Source: Clin Transl Gastroenterol. 2023 Dec 22;15(3):e00669. doi: 10.14309/ctg.0000000000000669 (PMC10962890; doi:10.14309/ctg.0000000000000669)
Supplement: Supplementary file 1 [file ct9-15-e00669-s001.docx]

**Supplemental table 1**: Response and remission at week 52 in patients with previous exposure to <2 vs ≥ 2 therapies with biologics.

| **Previous exposure to biologics** | | | |
| --- | --- | --- | --- |
|  | < 2  n=34 | ≥ 2  n=69 | *P*-value |
| **Response at week 52 (SCCAI < 5) n=44** | 47% | 41% | 0.5 |
| **Remission at week 52 (SCCAI ≤2) n=32** | 35% | 29% | 0.5 |
